# Pain-Related Coping Behavior in ALS: The Interplay between Maladaptive Coping, the Patient’s Affective State and Pain

**DOI:** 10.3390/jcm11040944

**Published:** 2022-02-11

**Authors:** Ina Schlichte, Susanne Petri, Reinhard Dengler, Thomas Meyer, Aiden Haghikia, Stefan Vielhaber, Susanne Vogt

**Affiliations:** 1 Department of Neurology, Otto-von-Guericke University, 39120 Magdeburg, Germany; ina.schlichte@gmx.de (I.S.); aiden.haghikia@med.ovgu.de (A.H.); stefan.vielhaber@med.ovgu.de (S.V.); 2 Department of Neurology, Diakonissenanstalt, 24939 Flensburg, Germany; 3 Department of Neurology, Hannover Medical School, 30625 Hannover, Germany; petri.susanne@mh-hannover.de (S.P.); dengler.reinhard@mh-hannover.de (R.D.); 4 Center for ALS and Other Motor Neuron Disorders, Department of Neurology, Charité-Universitätsmedizin, 13353 Berlin, Germany; thomas.meyer@charite.de; 5 Ambulanzpartner Soziotechnologie APST GmbH, 13353 Berlin, Germany; 6 German Center for Neurodegenerative Diseases (DZNE), 39120 Magdeburg, Germany; 7 Center for Behavioral Brain Sciences (CBBS), 39106 Magdeburg, Germany

**Keywords:** amyotrophic lateral sclerosis, motor neuron disease, pain, pain coping, depressive symptoms

## Abstract

Background: Pain is a common symptom in patients with amyotrophic lateral sclerosis (ALS). Coping plays a central role in adjustment to pain. Objective: This study evaluates the use of different pain coping strategies in patients with ALS and investigates the interplay of maladaptive coping, and the patient’s affective state and pain. Methods: One hundred and fifty ALS patients from three German outpatient clinics completed the Brief Pain Inventory (BPI), the ALS-Functional Rating Scale-Extension (ALSFRS-EX), the ALS Depression Inventory (ADI-12), the subscale “emotional functioning” of the ALS Assessment Questionnaire (ALSAQ-40) and the Coping Strategies Questionnaire (CSQ). Based upon the results of correlational analyses, multiple regression analyses were performed to identify predictors of pain severity and to explore factors contributing to maladaptive coping. Results: Pain was prevalent in 56% (*n* = 84) of the patients. Patients applied different adaptive coping strategies as well as the maladaptive strategy “catastrophizing”. Regression analysis indicated that the CSQ-subscale “catastrophizing” significantly predicted pain intensity, explaining 34.0% of the variance (*p* < 0.001). Pain-related catastrophizing was associated with higher pain-related functional impairments and worse emotional functioning. The ADI-12 sum score as an indicator for depressive symptoms contributed significantly to the maladaptive coping strategy “catastrophizing” (*p* < 0.001) and explained 40.8% of the variance. Conclusion: Patients with ALS apply different strategies to cope with pain. Catastrophizing is an important determinant of higher pain intensity ratings and is associated with higher pain interferences and decreased emotional well-being. Pain-related catastrophizing is promoted by depressive symptoms. Catastrophizing and depressive symptoms thus represent important targets of individualized pain-management strategies.

## 1. Introduction

Amyotrophic lateral sclerosis (ALS) is a devastating neurodegenerative disease marked by rapidly progressive loss of motor function. Patients develop rapidly progressive muscle weakness and wasting together with spasticity in limb and trunk muscles as well as bulbar symptoms, such as speech and swallowing dysfunction. Respiratory insufficiency and associated complications are the leading cause of patient death [1]. According to a recent German study, the median survival time is about 48 months [2]. Current clinical management mainly consists of symptomatic treatment to improve quality of life in patients [3].

A whole body of literature demonstrates that pain is a frequent and relevant symptom in ALS [4,5,6,7,8,9,10]. Even though pain has been suggested to increase throughout the course of the disease [4,11], it can also occur in the early stages [12]. Intensity and frequency of pain vary between studies [4,5,9,13], but there is consistent evidence that pain interferes with daily physical functions, such as general activity and work as well as emotional aspects, including joy of life and mood [4,5,14]. Pain thus represents an additional burden for patients who are already confronted with massive impairments in many areas of their lives. Studies demonstrated that pain is the most important contributor to suffering in patients with ALS [10] and has a negative impact on their quality of life [8,15]. The multifaceted impact of persistent pain requires the development of coping strategies.

In patients with chronic pain, coping behavior plays a central role in adaptation [16,17]. Coping behavior can be differentiated into active and passive coping styles [18]. While active coping is associated with the attempt to control pain, passive coping leaves the pain control to others [18,19]. Passive coping strategies have a negative impact on pain-related outcomes, which defines them as maladaptive [18,19,20,21]. A frequent passive coping strategy is “catastrophizing”, a negative pain-related mindset characterized by magnification of pain sensations, rumination and helplessness [16,22,23].

Coping in patients with ALS has been predominantly investigated in the context of adjustment to the disease itself [24,25], while pain-related coping has rarely been the subject of studies in ALS patients. One cross-sectional study on pain in ALS showed that pain intensity ratings correlated closely with “catastrophism” as a maladaptive coping strategy, as well as with anxiety and depressive symptoms. Of these factors, catastrophism had the biggest impact on the variability of pain intensity [26]. The interrelationship between catastrophizing and the patients’ affective state remained unclear. A qualitative study exploring presentation, consequences and management of pain in 16 patients with motor neuron disease revealed that successful coping behavior requires personal effort and competent engagement. However, this may impose an additional strain on patients [14]. This study further indicated that acceptance of pain and regulation of pain-related emotions play an important role in adjustment to pain in ALS, and suggests that an increased understanding of emotion-focused coping may enhance non-pharmacological treatment options in ALS [14].

Better understanding of factors influencing the experience of pain severity in patients with ALS and the different ways they adapt to pain could contribute to improvement of individual pain management. Analysis of a large cohort of ALS patients is necessary for thorough evaluation of pain-related coping behavior and to assess the relationship between pain, maladaptive coping strategies and the influence of a patient’s affective state.

The aim of our prospective multi-center study was the examination of different pain-related coping strategies and evaluation of the interplay between maladaptive coping, patient self-reported affective state and pain in a well-characterized cohort of ALS patients. We attempted to determine to what extent different coping strategies and the affective state can predict pain severity. In addition, the relationship between pain-related catastrophizing, pain-related impairments and emotional aspects of health-related quality of life as well as the influence of depressive symptoms on this maladaptive coping strategy should be investigated.

## 2. Methods

### 2.1. Study Design

The study was approved by the research ethics committee of the Medical Faculty of the University of Magdeburg and was part of a previous multi-center cross-sectional study on pain characteristics and treatment in patients with ALS [27]. The study was conducted between June 2015 and June 2019, and all patients provided written informed consent before enrollment. Patients with ALS aged 18 years or older were consecutively recruited by the ALS clinics at the university hospitals in Magdeburg, Hannover and Berlin. Exclusion criteria were patients with language barriers, which precluded their understanding of the questionnaires or severe cognitive impairment, as assessed by clinical judgment during the medical consultation. The final study cohort comprised 150 patients with ALS. All patients were contacted by telephone and provided with details of the study by our project team at the Department of Neurology at the University of Magdeburg.

### 2.2. Patient Characteristics

All patients were diagnosed with ALS based on the revised El Escorial criteria [28] and staged according to the onset of first symptoms as bulbar, upper or lower limb [29]. For clinical staging the King’s clinical staging system for ALS was used. It classifies the clinical impairment based on clinical milestones of the disease. The King’s stages were derived from the ALS Functional Rating Scale-Extension (ALSFRS-EX) and refer to the functional involvement of anatomical regions (stage 1 with one region involved up to stage 3 with a third region involved), and the need for nutritional support by provision of a percutaneous endoscopic gastrostomy (PEG) (stage 4A) and respiratory support via noninvasive ventilation (NIV) (stage 4B) [30,31].

### 2.3. Patient-Reported Data and Assessment Instruments

During the telephone contacts, we collected demographic and clinical data, such as region of symptom onset and disease duration, and asked questions about pain and pain medication being used in order to supplement the questionnaires.

In addition to the phone interviews, data were collected by means of questionnaires sent by mail to the patients. These questionnaires were all validated as patient-reported outcome measures in German. Physical functioning was evaluated by the disease-specific ALS Functional Rating Scale-Extension (ALSFRS-EX). This scale comprises 15 items divided into four subscales (i.e., bulbar, fine motor, gross motor and respiratory) [32,33]. The sum score ranges from 0 to 60 points with a maximum subscale score of 16 points for the bulbar, fine and gross motor subscales and 12 points for the respiratory subscale. Lower scores represent a worse condition.

Pain was evaluated with the Brief Pain Inventory (BPI)—Long Form [34]. The BPI provides information on the intensity of pain as well as its interference with daily functioning. The respective rating scales reach from 0 to 10 using a recall period of the past week. In addition, the BPI uses anatomic drawings of the front and back human body where the patient can mark the location of his pain.

Pain coping behavior was assessed by the Coping Strategies Questionnaire—German version (CSQ-D) [35,36]. The questionnaire consists of 48 items that evaluate eight coping strategies (coping self-statements, increasing pain behavior, ignoring pain sensations, increasing activity level, diverting attention, reinterpreting pain sensations, catastrophizing, praying or hoping). Answers were given on a 7-point Likert scale from 0 (“never do that”) to 6 (“always do that”). The items 16 “I walk a lot” and 21 “I try to think years ahead, what everything will be like after I have gotten rid of the pain” were left out because of possible interferences with ALS-related physical impairment and the limited life expectancy. The mean score for each subscale was computed. The coping strategies were further classified into an active and passive coping style [18].

Further questionnaires referred to the patient’s self-reported affective state in terms of depressive symptoms and emotional well-being as a subdimension of health-related quality of life. Therefore, patients were asked to complete the ALS Depression Inventory (ADI-12) as a measure for depressive symptoms [37] and the subscale “emotional functioning” of the ALS Assessment Questionnaire (ALSAQ-40) [38], which are both disease-specifically developed assessment instruments.

The ALS Depression Inventory (ADI-12) includes 12 questions, which are answered on a scale from 1 (“I fully agree”) to 4 (“I do not agree at all”). A sum score below 22 points indicates the absence of depression, 22 to 28 points mild depressiveness and above 28 points clinically relevant depressive symptoms.

The ALS Assessment Questionnaire (ALSAQ-40) refers to health-related quality of life in ALS patients and consists of 40 items, which are classified into 5 domains (“eating and drinking”, “communication”, “activity of daily life/independency”, “physical mobility” and “emotional functioning”). For this study, only the subscale “emotional functioning” (10 items) was analyzed. Each item is rated on a 0 (never) to 4 (“always/cannot do at all”) scale. The values of the subscale were transformed into a range from 0 to 100 ((sum score of the 10 items of the subscale “emotional functioning”/40) × 100). A lower score represents a better emotional well-being [38].

Questionnaires were completed by the patients or, if this was not possible due to severe functional impairment, by a family member or caregiver at the patient’s instructions and then returned.

### 2.4. Statistics

Statistical analysis was conducted using IBM SPSS, Version 24 (IBM Corp., Armonk, NY, USA). Demographic characteristics and clinical data were summarized using descriptive statistics (frequencies (%) or mean and standard deviation, as appropriate).

Bivariate correlations were conducted to examine associations between study variables (demographic, clinical and pain-related variables as well as the ALSFRS-EX sum score, ADI-12 sum score, subscale scores of the CSQ and ALSAQ-40 subscale “emotional functioning”). They were calculated using the Pearson or Spearman coefficient for normally or non-normally distributed data, respectively. Correlations between nominal and interval variables were analyzed with the Eta coefficient. For interpretation of effect sizes, correlation values between 0.10 and 0.29 were considered weak, from 0.30 to 0.49 moderate, and from 0.50 to 1.00 strong [39]. Comparisons between the groups were performed using Chi square test or Fisher’s exact test, as appropriate, for categorical data and the independent samples t-test or the Mann–Whitney U test were used for continuous data depending on the distribution of the data.

A multiple regression analysis was performed to identify predictors of the average pain intensity according to the BPI. After catastrophizing emerged as a predictor of pain severity, we then performed a second regression analysis with catastrophizing as the dependent variable. The significance level was set at α ≤ 0.05, unless stated otherwise, and corrected for multiple comparisons using Bonferroni adjustments, if necessary.

## 3. Results

### 3.1. Demographic and Clinical Data

One hundred and fifty patients participated in the study, of whom eighty-four patients reported pain. Hence, the prevalence of pain was 56%. Patient-referred data of the patients with and without pain are presented in Table 1.

Patients with pain were significantly younger than patients without pain (*p* = 0.037) and had significantly more physical impairments reflected by a lower total score of the ALSFRS-EX (*p* = 0.003). Referring to the self-reported affective state, patients with pain scored significantly higher on the ADI-12 (*p* = 0.002) and the emotional subscale of the ALSAQ-40 (*p* = 0.003), indicating a higher level of depressive symptoms and worse emotional functioning. No significant differences were found concerning other demographic and clinical data.

### 3.2. Pain Characteristics, Treatment and Pain Relief

Of the 84 patients with pain, the mean score for the average pain in the past week was 4.0 ± 1.9 on the NRS, indicating a moderate pain intensity level. The mean NRS score for the most severe pain in the past week was 5.5 ± 2.0. Pain occurred across all stages of the disease. The lumbar region, neck, shoulder region, calf (>30%) and the buttocks and the proximal leg (>20%) were most commonly affected. Pain interfered with all functions of daily living, which is summarized in Table 2. Fifty-four of the pain patients received analgesic medication (64.3%). An overview of the medication in use is presented in Table 2. More details about the pain characteristics and treatment have been reported elsewhere [27].

### 3.3. Pain-Related Coping Strategies

**Figure 1:**
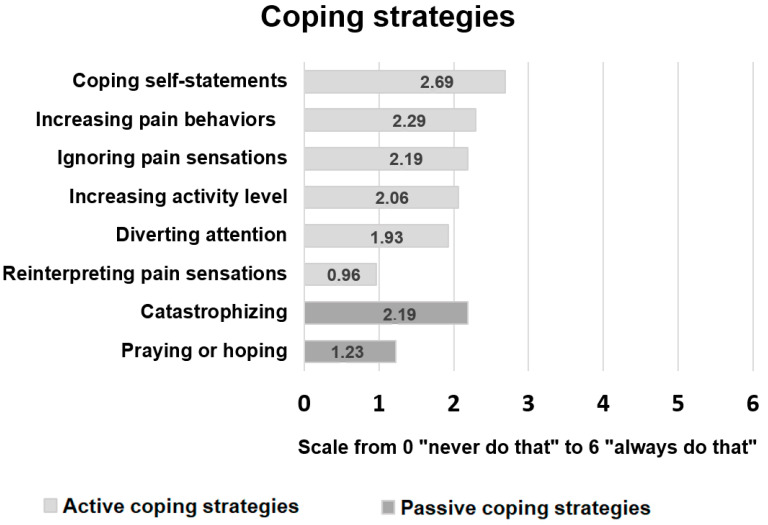
Use of pain coping strategies in ALS patients with pain, indicating the mean values of the CSQ subscale scores on a scale from 0 “never do that” to 6 “always do that”. The different pain coping strategies are grouped into active (highlighted in light grey) and passive coping strategies (highlighted in grey) and are arranged in descending order of the mean subscale scores within the respective category of coping style.

The patients’ use of the different pain-related coping strategies is shown in Figure 1. The most frequently used active coping strategies were “coping self-statements” (2.69 ± 1.39) and “increasing pain behaviors” (2.29 ± 1.21) followed by “ignoring pain sensations” (2.19 ± 1.35). With regard to passive coping, “catastrophizing” (2.19 ± 1.59) was clearly more often used than “praying or hoping” (1.23 ± 1.18).

### 3.4. Relation between Demographic/Clinical Data, Pain-Related Features and the Affective State

**Figure 2:**
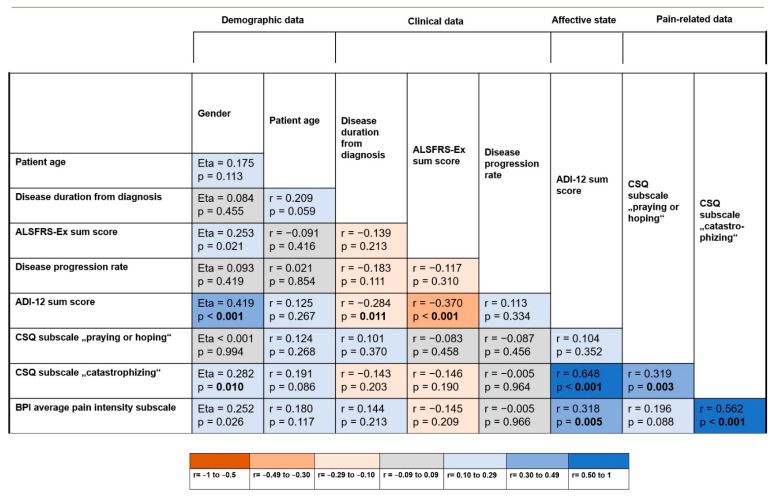
Correlation matrix presenting the correlations between demographic and clinical data as well as the results of the patient-reported outcome measures referring to the ALS patients with pain. For visualization of the degree of the association between the variables, a color scale from shades of red to shades of blue was used, which refers to the interpretation of effect sizes in terms of weak (0.10 to 0.29), moderate (0.30 to 0.49) and strong (0.50 to 1.00). Referring to the four groups of variables (demographic data, clinical variables, measure of affective state and pain-related variables), Bonferroni-adjusted *p*-values: 0.05/4 = 0.0125 were deemed significant and are boldfaced.

The degree of associations between demographic and clinical variables, pain-related data and the affective state (ADI-12 sum score) is visualized in a correlation matrix in Figure 2. 

Strong correlations were found between the CSQ subscale score for “catastrophizing” and the average pain intensity (r = 0.562, *p* < 0.001) as well as the ADI-12 sum score (r = 0.648, *p* < 0.001). Highly significant correlations on a moderate level were found between the ADI-12 sum score and the average pain intensity (r = 0.318, *p* = 0.005) as well as gender (Eta = 0.419, *p* < 0.001). The CSQ subscale scores for the maladaptive coping strategies “catastrophizing” and “praying or hoping” correlated with each other on a moderate level (r = 0.319, *p* = 0.003).

A moderate inverse correlation was found between the ALSFRS-EX sum score representing the functional status and the ADI-12 sum score (r = −0.370, *p* < 0.001), indicating a higher level of depressive symptoms in patients with higher physical impairment.

### 3.5. Predictors of the Average Pain Intensity

Regarding the correlations between ratings for the mean pain intensity and pain-related coping strategies, a strong correlation was found for the maladaptive coping strategy “catastrophizing” (r = 0.562, *p* ≤ 0.001) and a moderate correlation was found for the adaptive coping strategy “increasing pain behaviors” (r = 0.374, *p* = 0.001). A weak association was found with the CSQ subscale “diverting attention” (r = 0.244, *p* = 0.032).

Referring to the ADI-12, a significant correlation with the average pain intensity on a moderate level was found (r = 0.318, *p* = 0.005).

Regarding the demographic variables of age and gender and the clinical variables of disease duration from diagnosis, ALSFRS-EX sum score and disease progression rate, there was a weak inverse association between the average pain intensity and gender (r = −0.252, *p* = 0.026).

A multiple regression analysis (method: enter) was executed with all parameters, which correlated significantly with the average pain intensity, to identify potential predictors. An overview of the results is provided in Table 3.

The results of this regression analysis showed that the model was a significant predictor of the average pain intensity (F (5, 70) = 8.731, *p* < 0.001). While the CSQ subscale score for “catastrophizing” contributed significantly to this model (ß = 0.529, *p* < 0.001), all of the other variables did not. The coping strategy of “catastrophizing” was able to explain 34.0% of the variance of the average pain intensity (adjusted R^2^ = 0.340, *p* < 0.001).

**Figure 3:**
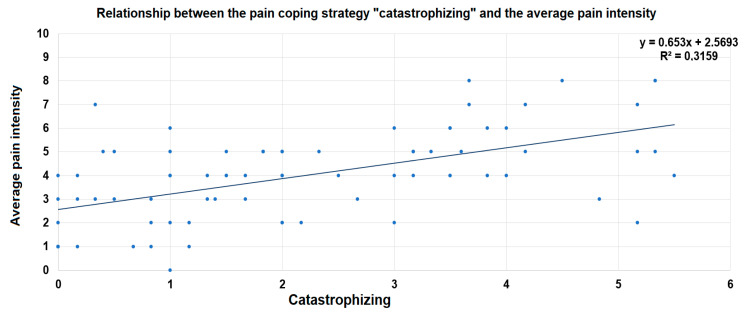
Scatter plot of the relationship between the CSQ subscale score for “catastrophizing” and the average pain intensity ratings according to the BPI. A linear regression line has been superimposed.

Figure 3 illustrates the relationship between the CSQ subscale score for “catastrophizing” and the average pain intensity ratings. Accordingly, patients with a higher use of the maladaptive coping strategy “catastrophizing” tended to experience higher average pain intensity.

### 3.6. Catastrophizing in Relation to the Patients’ Functional Status and Emotional Aspects of Health-Related Quality of Life 

The CSQ subscale score of “catastrophizing” correlated significantly with five out of seven pain-related impairments of daily functions listed in the BPI (Bonferroni-adjusted *p*-values: 0.05/7 = 0.007). Strong correlations were observed for “mood” (r = 0.661, *p* < 0.001), “relations with other people” (r = 0.550, *p* < 0.001), “sleep” (r = 0.520, *p* < 0.001) and “enjoyment of life” (r = 0.675, *p* < 0.001) and a correlation on a moderate level for “general activity” (r = 0.431, *p* < 0.001).

Regarding the ALSAQ-40 subscale for “emotional functioning”, there was a strong correlation with “catastrophizing” (r = 0.589, *p* < 0.001).

### 3.7. Predictors of Pain-Related Catastrophizing

When correlating the CSQ subscale score of “catastrophizing” with the demographic variables age and gender and the clinical variables disease duration from diagnosis and disease progression rate as well as with the ADI-12 sum score, there was a strong and highly significant correlation with the ADI-12 sum score (r = 0.648, *p* < 0.001) and a weak correlation with gender (Eta = 0.282, *p* = 0.010).

Subsequently, a multiple regression analysis (method: enter) was undertaken to see if these variables (ADI-12 sum score and gender) predict the use of the coping strategy “catastrophizing”.

The findings of this regression analysis showed that the model was a significant predictor to the coping strategy “catastrophizing” (F (2, 79) = 28.875, *p* < 0.001) and accounted for 40.8% of the variance (adjusted R^2^ = 0.408). Apart from the ADI-12 sum score, which was a significant contributor to this model (ß = 0.625, *p* < 0.001), gender did not significantly contribute to it. Variance inflation factor values for both variables in the model were 1.213.

**Figure 4:**
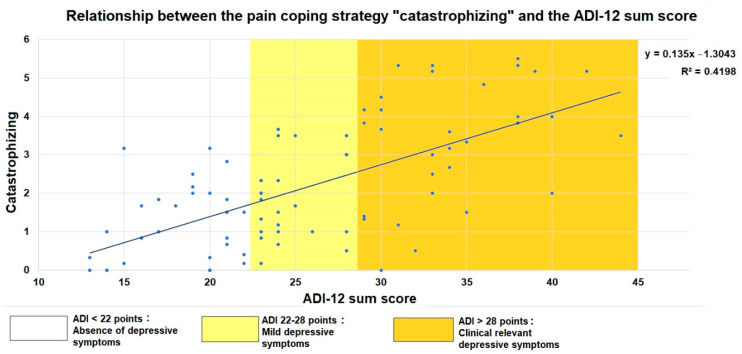
Scatter plot of the relationship between the CSQ subscale score for the coping strategy of “catastrophizing” and the ADI-12 sum score as a measure of depressive symptoms. A linear regression line has been superimposed.

The relationship between the coping strategy of “catastrophizing” and the ADI-12 sum score is illustrated in Figure 4. Hence, higher scorings for depressive symptoms go along with a more frequent use of the maladaptive coping strategy “catastrophizing”.

## 4. Discussion

### 4.1. Pain-Related Coping Behavior

Our study demonstrated that ALS patients with pain use a variety of coping strategies. The most frequently used coping strategies were “coping self-statements” and “increasing pain behaviors”, both representing an active coping style. Among the passive coping strategies, “catastrophizing” was used far more frequently than “praying or hoping”.

Our finding that ALS patients with pain use different coping strategies is in line with a recent qualitative study from Sweden on the experience, the consequences and management of pain in patients with ALS [14]. While Åkerblom et al. performed interviews in 16 patients with ALS to inquire the individual pain management, we used the Coping Strategies Questionnaire as a comprehensive patient-reported instrument. This approach allowed for a standardized evaluation of different pain-related coping strategies in a comparatively large cohort of ALS pain patients.

Our observation that the active coping strategies “coping self-statements” and “increasing pain behaviors” were used most frequently and “reinterpretation of pain sensations” was used least frequently is consistent with the findings of other studies on patients with chronic non-malignant pain conditions, such as fibromyalgia syndrome, rheumatoid arthritis, peripheral neuropathic pain and chronic low back pain [35,41,42]. However, the comparability of pain adaptation in these chronic pain conditions to pain adaptation in a disease as devastating as ALS is limited as ALS patients may experience more emotional distress due to the fatal nature of the disease [43,44], which may, in turn, have a negative impact on their psychological resources for coping with pain.

According to our study, ALS patients with pain frequently used pain-related catastrophism. This corresponds to the findings of a cross-sectional study on pain in ALS, which evaluated the impact of catastrophism, depression and anxiety on pain severity [26]. In contrast to this study, in which catastrophism was merely assessed as one single possibility of coping, our study evaluated “catastrophizing” as one of several other pain-related coping strategies by the CSQ and thus within a superordinated concept of pain coping. This allowed for further insights into pain-related coping behavior in ALS patients.

### 4.2. Interplay between Pain, Maladaptive Coping and the Patients’ Affective State

Our study demonstrated that the maladaptive coping strategy of “catastrophizing” has important clinical implications, as catastrophizing was associated with higher pain severity and pain interference as well as lower emotional well-being. This maladaptive mode of pain adaptation explained 34.0% of the variability of pain severity in patients with ALS indicating an association of pain-related catastrophizing with higher levels of pain severity. Further, our study showed that self-reported depressive symptoms did not have a direct influence on the experience of pain severity. However, there was a close relationship between depressive symptoms and pain-related catastrophism. Accordingly, the ADI-12 sum score was the main determinant of pain-related catastrophizing explaining 40.7% of the variance.

Corresponding to our results, a previous study found that pain-related catastrophism is the most relevant factor for the prediction of the pain intensity in ALS [26]. This study further suggested that depression and anxiety interact with pain severity. Even though we also observed highly significant correlations between pain severity and depressive symptoms in our initial correlation analysis, this association could not be confirmed in the regression model in which the maladaptive coping strategy of catastrophizing was also considered. This inconsistency between our findings and those of Lopes et al. regarding the relationship between pain severity, depressive symptoms and catastrophism may be attributed to the use of different assessment instruments. The Hospital Anxiety and Depression Scale used by Lopes et al. contains items referring to symptoms of depressiveness that can, in patients with ALS, also be affected by the underlying disease. For instance, the items “I can sit at ease and feel relaxed” and “I can laugh and see the funny side of things” can be influenced by ALS-related impairments of gross motor and bulbar function rather than the presence of depressive symptoms. In our study, we therefore used the ADI-12 as a disease-specific and validated measure that excludes items that may interfere with ALS-related impairments. Furthermore, both studies assessed pain-related catastrophizing in different ways. In our study, catastrophizing was evaluated by the respective subscale of the Coping Strategies Questionnaire, whereas Lopes et al. used a single item measure, the so-called Pain-Related Catastrophizing Thoughts Scale [26].

The literature on the association between pain in ALS and depressive symptoms is inconsistent [13,26,45,46,47]. A recent study on this topic summarized that pain severity and depressive symptoms interfere with different domains of a patient’s quality of life but do not necessarily determine each other [8]. This interpretation is supported by our study results.

Further insights into the interplay between pain, catastrophizing and depressive symptoms can be gained from previous studies in chronic pain patients. Pain-related catastrophism was found to be associated with higher levels of pain severity in patients with different chronic pain conditions [41,48,49,50,51,52].

A prior study, which investigated the relationship between catastrophizing, depressive symptoms and pain in chronic pain patients; however, provides results that are partially contradictory to our findings [53]. The greatest association with catastrophizing as measured by the CSQ was seen for negative mood, specifically depressive symptoms, with little link to pain. The authors suppose that even though catastrophizing is related to negative mood, it is a unique construct that is distinct from negative mood. They discuss that the strong associations of the CSQ subscale catastrophizing with negative mood scores may be a limitation of the clinical utility of this subscale but may also endorse its potential as a brief outcome measure in certain applications, e.g., in a treatment program aimed at improving the patient’s coping skills and psychological well-being.

In our study, depressive symptoms also predicted a relevant proportion of the variance of catastrophizing. The main difference to the study findings of Hirsh et al. is that depressive symptoms did not predict pain severity in our ALS patients with pain, but catastrophizing predicted a relevant proportion of the variance in pain severity despite accounting for depressive symptoms. There was no relevant multicollinearity between catastrophizing and depressive symptoms, which implies that both constructs are indeed distinct.

However, the two studies are not directly comparable because different assessment instruments for depressive symptoms and pain were used and the study cohorts differed (chronic pain patients vs. a fatal neurological disorder).

Another study in chronic pain patients may allow for a more nuanced understanding of the complex interrelationships between pain, catastrophizing and depressive symptoms [54]. According to this study, catastrophizing mediates the association between depressive symptoms and the affective and evaluative experience of pain, whereas depressive symptoms are directly related to the sensory aspect of pain. The authors thus suggest that catastrophizing may play a greater role than depressiveness when it comes to evaluation of pain experience [54]. The latter is supported by our study findings.

Our finding that catastrophizing is associated with higher pain interference, decreased emotional well-being and more intense pain and is in turn influenced by depressive symptoms, may have important implications for the treatment of pain in ALS. It offers the possibility to optimize individual pain treatment strategies to reduce maladaptive coping and to enable patients to use adaptive coping skills. Given that successful coping is based upon high personal effort and engagement [14], the suitability of such interventions should be carefully evaluated on an individual basis. Further research on the effectiveness of behavioral therapies in ALS patients with pain may help to better balance potential benefits and unreasonable additional burdens.

## 5. Limitations

There are several study limitations. One possible limitation of this study is the enrolment of patients from university-based outpatient clinics. This may have implications to the generalizability of our findings in that these patients may have been more proactive about their own care and pain management. Presumably, they may have also been more motivated to develop adaptive coping strategies. This may be a bias, which is intrinsic to investigations performed at specialized outpatient clinics.

Further, the recruitment of patients from three ALS outpatient clinics raises the question of variability in results between the centers. Another potential limitation refers to the sample size of our study. Although this is relatively large given the rarity of this disease, an even larger study cohort would be warranted to allow for subgroup analyses of pain coping in different ALS subtypes.

## 6. Conclusions

Patients with ALS apply different strategies to cope with pain. The maladaptive coping strategy of catastrophizing is an important determinant of higher pain intensity ratings and is associated with higher pain interferences and decreased emotional well-being. Pain-related catastrophizing is promoted by depressive symptoms. Catastrophizing and depressive symptoms thus represent important targets of individualized pain-management strategies and may offer a possibility to enhance the quality of life in patients.

## Figures and Tables

**Table 1:** Demographic and clinical data as well as results from patient reported outcomes of ALS patients with and without pain (mean ± SD or number of patients (%), as appropriate).

	Patients with Pain	Patients without Pain	*p*-Value
Gender (female/male)	24/60 (28.6/71.4%)	20/46 (30.3/69.7%)	0.817
Patient age	61.2 ± 11.8	66.6 ± 8.0	**0.037**
Disease duration from diagnosis in months	33.5 ± 38.5	37.7 ± 51.3	0.569
Symptom onset			0.135
Bulbar	14 (16.7%)	19 (28.8%)	
Upper limb	37 (44%)	21 (31.8%)	
Lower limb	31 (36.9%)	22 (33.3%)	
Missing information	2 (2.4%)	4 (6.1%)	
King’s clinical staging			0.065
Stage 1: Symptom onset/functional involvement of first region	0	0	
Stage 2A: Diagnosis	7 (8.3%)	5 (7.6%)	
Stage 2B: Functional involvement of a second region	8 (9.5%)	16 (24.2%)	
Stage 3: Functional involvement of a third region	39 (46.4%)	23 (34.8%)	
Stage 4A: Need for gastrostomy	4 (4.8%)	7 (10.6%)	
Stage 4B: Need for respiratory support (NIV)	26 (31%)	15 (22.7%)	
ALSFRS-EX			
Sum score	36.6 ± 12.9	42.6 ± 10.8	**0.003**
Bulbar subscore	11.6 ± 4.8	11.23 ± 3.4	0.344
Fine motor subscore	7.7 ± 4.3	10.0 ± 4.9	**0.003**
Gross motor subscore	8.2 ± 5.0	10.2 ± 4.6	**0.012**
Respiratory subscore	9.1 ± 3.2	10.2 ± 2.8	0.038
Disease progression rate *	0.9 ± 1.9	0.75 ± 0.9	0.639
ADI-12			
Sum score	26.1 ± 7.6	22.2 ± 7.1	**0.002**
Absence of depressive symptoms (<22 points)	25 (29.8%)	33 (50%)	**0.029**
Mild depressive symptoms (22–28 points)	26 (31%)	18 (27.3%)	
Clinically relevant depressive symptoms (>28 points)	31 (36.9%)	14 (21.2%)	
ALSAQ-40 subscale score “emotional functioning”	41.8 ± 22.6	30.9 ± 20.2	**0.003**

NIV: Non-invasive ventilation; ALSFRS-EX: ALS Functional Rating Scale-Extension; ALSAQ: Amyotrophic Lateral Sclerosis Assessment Questionnaire; ADI: ALS Depression Inventory. * Disease progression rate was calculated as (60-sum of ALSFRS-EX)/disease duration from symptom onset to investigation date in months (adapted from [33,40]). Significant *p*-values are boldfaced. Referring to the ALSFRS-EX subscales, Bonferroni-adjusted *p*-values 0.05/4 = 0.0125 were deemed statistically significant. Some of these data were published in a previous publication from this study, see [27].

**Table 2:** Data referring to the patients’ ratings of pain interference with daily functions and the medication-related pain relief. Values are given as mean ± SD or number of patients (%), as appropriate.

	Patients with Pain (*n* = 84)
Pain interference with daily functions	
Normal work	5.4 ± 3.0
Walking ability	4.8 ± 2.8
General activity	4.5 ± 3.2
Sleep	4.0 ± 3.7
Mood	4.2 ± 3.1
Enjoyment of life	3.6 ± 3.7
Relations with other people	2.9 ± 3.1
Pain-related pharmacotherapy *	*n* = 54 (64.3%)
Non-opioid analgesics	*n* = 45 (53.6%)
Opioid analgesics	*n* = 16 (19.0%)
Tricyclic antidepressants **	*n* = 5 (6.0%)
Anticonvulsants	*n* = 10 (12.0%)

* This table does not consider any combination of therapies. Thus, the total percentage of patients is higher than 100%. ** Antidepressants refer to their use for analgesic purposes.

**Table 3:** Multiple regression analysis of potential predictors of the average pain intensity ratings according to the Brief Pain Inventory (dependent variable).

	Unstandardized Coefficients		Standardized Coefficients	T	*p*-Value	VIF
	Regression Coefficient B	Standard Error	Beta			
Gender	−0.521	0.465	−0.119	−1.120	0.266	1.279
CSQ subscales						
Diverting attention	0.323	0.170	0.216	1.899	0.062	1.471
Catastrophizing	0.621	0.160	0.529	3.873	**<0.001**	2.121
Increasing pain behaviors	0.086	0.196	0.054	0.439	0.662	1.699
ADI-12 sum score	−0.019	0.033	−0.078	−0.593	0.555	1.973

Significant *p*-values are boldfaced. VIF = variance inflation factor.

## Data Availability

Data are contained within the article.
